# Impact of diabetes on sarcopenia and mortality in patients undergoing hemodialysis

**DOI:** 10.1186/s12882-019-1271-8

**Published:** 2019-03-28

**Authors:** Katsuhito Mori, Kozo Nishide, Senji Okuno, Tetsuo Shoji, Masanori Emoto, Akihiro Tsuda, Shinya Nakatani, Yasuo Imanishi, Eiji Ishimura, Tomoyuki Yamakawa, Shigeichi Shoji, Masaaki Inaba

**Affiliations:** 1 Department of Nephrology, Osaka City University Graduate School of Medicine, 1-4-3 Asahi-machi, Abeno-ku, Osaka, 545-8585 Japan; 2 Department of Metabolism, Endocrinology and Molecular Medicine, Osaka City University Graduate School of Medicine, Osaka, Japan; 3 Kidney Center, Shirasagi Hospital, Osaka, Japan; 4 Department of Vascular Medicine, Osaka City University Graduate School of Medicine, Osaka, Japan

**Keywords:** Sarcopenia, Diabetes mellitus, Hemodialysis, Chronic kidney disease

## Abstract

**Background:**

Sarcopenia has become a serious disorder in modern society. Chronic kidney disease requiring dialysis and diabetes are some of the disorders that accelerate the onset and progression of sarcopenia. We, therefore, investigated the prevalence of sarcopenia in patients undergoing hemodialysis (HD) and confirmed the impact of diabetes mellitus (DM) on this population.

**Methods:**

This study included 308 patients whose muscle strength and mass had been evaluated using handgrip strength and dual-energy X-ray absorptiometry, respectively. Sarcopenia was defined according to the criteria established by the Asian Working Group on Sarcopenia. In addition, this cohort had been followed up for 9 years.

**Results:**

The prevalence of sarcopenia was 40% (37% in males and 45% in females) with gender differences being insignificant (*p* = 0.237). The DM morbidity rate was significantly higher in those with sarcopenia than in those without sarcopenia (41% vs. 27%, *p* = 0.015). Multivariate regression analyses showed that the presence of DM was an independent contributor to sarcopenia in patients undergoing HD (odds ratio 3.11; 95% confidence interval 1.63–5.93; *p* <  0.001). During the follow-up of 76 ± 35 months, 100 patients died. Patients with sarcopenia demonstrated significantly higher rates of all-cause mortality than those without sarcopenia (p <  0.001 using the log-rank test). Multivariate Cox proportional hazards analyses revealed that the presence of DM was significantly associated with higher all-cause mortality (adjusted hazard ratio: 2.39; 95% confidence interval 1.51–3.81; p <  0.001).

**Conclusions:**

The prevalence of sarcopenia among this cohort of patients undergoing HD was determined to be 40%. Moreover, the presence of DM was an independent contributor to sarcopenia and an independent predictor of all-cause mortality in this population.

**Electronic supplementary material:**

The online version of this article (10.1186/s12882-019-1271-8) contains supplementary material, which is available to authorized users.

## Background

Although medical advancements and improved healthcare have resulted in increased longevity, particularly in developed countries, they have resulted in unexpected and serious conditions, such as sarcopenia. Sarcopenia has been generally defined as the loss of skeletal muscle mass and function, resulting in poor outcomes mainly through physical impairment. Although the concept of sarcopenia is clear, its current status in clinical practice is unclear. One of the major reasons may be the lack of an exact definition for sarcopenia. In response, the European Working Group on Sarcopenia in Older People first reported a consensus on the definition of sarcopenia, which involved muscle mass, strength, and/or physical function [1]. Moreover, criteria for the Asian population have been recently established by the Asian Working Group on Sarcopenia (AWGS) in 2014 with consideration for ethnic variation [2].

In addition to inevitable aging, some chronic conditions have been well-known to modulate and exacerbate sarcopenia. Among them, chronic kidney disease (CKD), particularly that requiring dialysis, is a disorder that accelerates the symptoms of sarcopenia [3, 4]. However, conflicting results exist concerning its precise prevalence. Sarcopenia has been reported to be present in 20% of European patients undergoing dialysis (mean age: 53 ± 13) [5] whereas it has a higher prevalence in Asian patients undergoing hemodialysis (HD) (37.0% in men and 29.3% in women) (mean age: 63.9 ± 10.0) [6]. Even among Asian patients undergoing HD, much lower incidences of sarcopenia (13.7%) have been reported (mean age: 49.4 ± 11.7) [7]. These findings suggest that various definitions of sarcopenia could lead to its different prevalence in addition to disparity in age.

Diabetes mellitus (DM), another highly prevalent disorder in modern society, has been reported to be associated with sarcopenia [8] [9]. Recently, using the AWGS criteria, Wang et al. demonstrated, for the first time, that the prevalence of sarcopenia was significantly higher in patients with DM than in healthy subjects (14.8% vs. 11.2%) [10].

To establish appropriate countermeasures for the aging society, it becomes imperative to determine accurate figures for sarcopenia using uniform criteria in consideration of ethnicities, body size, life style, and cultural backgrounds [2], particularly in populations prone to requiring nursing care. Especially, advanced CKD including HD is one of representative disorders that show malnutrition, body weight loss, and concomitant physical dysfunction independent of age [3, 4]. Accurate figures for sarcopenia are necessary to assess the benefits of intervention or to compare the morbidity rate of sarcopenia among different cohorts. In the present study, we first examined the prevalence of sarcopenia in patients undergoing HD using the AWGS criteria while considering the involvement of DM, the most common primary disease associated with HD. We then investigated whether the presence of sarcopenia as well as DM could predict mortality in this population.

## Methods

### Participants and data collection

A total of 308 patients undergoing HD at Shirasagi Hospital were included in this study. Patients underwent HD three times a week on Monday, Wednesday, and Friday. The diagnosis of DM was based on a history of DM or the American Diabetes Association criteria [11]. The main exclusion criteria included HD treatment for < 1 year or > 21 years, malignant tumors, active inflammatory diseases, systemic lupus erythematosus, rheumatoid arthritis, chronic obstructive pulmonary disease, and a history of tuberculosis and amputation. All participants could ambulate without assistance. This study was conducted in accordance with the principles of the Declaration of Helsinki. Informed consent was obtained from each patient. Since the cohort wasn’t originally for this study, modified protocol was retrospectively approved by the ethics committee of Shirasagi Hospital (registration number J2017015). Since the participant consent was verbal, approval for an opt-out consent method was given. Blood sampling was performed immediately before starting the Monday HD session, which was exactly 68 h after the previous (i.e., Friday) session. Blood samples were collected from the arteriovenous fistula or graft.

### Diagnosis of sarcopenia

Muscle strength was assessed through handgrip strength (HS) using a hand dynamometer (Smedley type hand dynamometer; AS ONE corporation, Japan) by experienced research staff who were blinded to all clinical and biochemical data, as reported previously [12, 13]. More precisely, HS was measured alternately in each hand once before or after HD. Patients were instructed to hold the grip with maximum force, with the arm extended, in the upright position. The higher value was recorded.

Muscle mass was measured through dual-energy X-ray absorptiometry (DXA). Each participant was weighed and then underwent whole-body DXA scanning (QDR-1000 W; Hologic Inc., Waltham, MA, USA) 21–24 h after completing the dialysis session [12, 13]. Fat-free mass (lean mass) was calculated by subtracting fat mass from the “dry weight”. Skeletal mass index (SMI) (kg/m^2^) was calculated by dividing the appendicular skeletal muscle mass by the body height in meters squared [2].

We adopted the AWGS criteria for diagnosis of sarcopenia [2]. AWGS recommends strategy for sarcopenia screening and assessment by dividing cases into 2 categories (ie, community settings and specific chronic conditions in all health care settings). As the screening test for community-dwelling older people, measuring of both handgrip strength and gait speed are recommended. Since the cohort in this study belongs to chronic conditions (eg, chronic kidney disease, diabetes mellitus) defined by AWGS, we used the cutoff values recommended by AWGS for muscle strength and muscle mass measurements as follows. Low muscle strength was defined as HS < 26 and 18 kg for men and women, respectively. Low muscle mass by using DXA was defined as SMI < 7.0 and 5.4 kg/m^2^ for men and women, respectively. Diagnosis of sarcopenia was established in patients presenting with both low muscle strength and mass as per the AWGS criteria [2].

### Mortality data collection

The cohort was monitored until the end of December 2005 with a mean ± standard deviation (SD) and median [interquartile range (IQR)] follow-up of 76 **±** 35 and 90 (44–108) months, respectively. Observational period of this study was from January 1997 to December 2005. At the end of the follow-up, 131 patients were confirmed to have survived, whereas 100 patients died. The remaining 77 patients had transferred to another hospital during the follow-up.

### Statistical analyses

Data have been expressed as numbers, percentages, mean ± SD, or median (IQR) as appropriate. Proportions have been expressed as percentages. Differences in mean, median, and percentages between the two groups were evaluated using Student’s t-test, Mann–Whitney U test, and χ^2^ test, respectively. Survival curves were constructed using the Kaplan–Meier method and evaluated using the log-rank test. Prognostic variables were examined using the multivariate Cox proportional hazards model, with hazard ratios (HRs) and 95% confidence intervals (CIs) being calculated. A *p* value of 0.05 was considered to be statistically significant. Statistical analysis used StatView 5 system (SAS system, Cary, NC) for Windows.

## Results

### Participant characteristics

Table 1 shows clinical and biochemical profiles of participants included in this study. The prevalence of sarcopenia was determined to be 40% (37% in males and 45% in females) with no significant differences based on gender (*p* = 0.237). Age and C-reactive protein (CRP) levels, and Kt/V were significantly higher in patients with sarcopenia than in those without sarcopenia. On the other hand, serum albumin, blood urea nitrogen (BUN), creatinine, and phosphate levels were significantly lower in those with sarcopenia than in those without sarcopenia. The DM morbidity rate was significantly higher in those with sarcopenia than in those without sarcopenia (41% vs. 27%, *p* = 0.015). No differences in hemoglobin levels, duration of HD, and normalized protein catabolic rate (nPCR) were observed between the groups.

**Table 1:** Characteristics of study participants

	Without sarcopenia	With sarcopenia	p
Number of participants (%)	184 (60%)	124 (40%)	
Gender (male/female)	116 (38%)/68 (22%)	69 (22%)/55 (18%)	0.237
Age (y)	54.4 ± 11.0	63.5 ± 11.0	< 0.001
Duration of HD (y)	6.0 ± 5.6	7.1 ± 6.7	0.213
BMI (kg/m2)	21.2 ± 2.8	19.4 ± 2.5	< 0.001
Diabetes (%)	50 (27%)	51 (41%)	0.015
Serum Albumin (g/dL)	4.1 ± 0.3	3.9 ± 0.3	< 0.001
BUN (mg/dL)	83.5 ± 13.6	78.4 ± 12.2	0.001
Serum Creatinine (mg/dL)	11.9 ± 2.6	9.9 ± 2.2	< 0.001
Phosphate (mg/dL)	6.2 ± 1.3	5.7 ± 1.1	< 0.001
Hemoglobin (g/dL)	8.9 ± 1.2	8.9 ± 1.2	0.848
HS (kg)	28.2 ± 9.6	15.7 ± 6.3	< 0.001
SMI (kg/m2)	6.17 ± 1.03	5.13 ± 0.72	< 0.001
CRP (mg/dL)	0.15 (0.10–0.46)	0.20 (0.12–0.72)	0.018
Kt/V	1.12 ± 0.24	1.20 ± 0.27	0.009
nPCR (g/kg per day)	1.05 ± 0.22	1.03 ± 0.21	0.662

Data are expressed as numbers, percentages, mean ± standard deviation, or median (interquartile range)

Abbreviations: *H* hemodialysis, *BMI* body mass index, *BUN* blood urea nitrogen, *HS* handgrip strength, *SMI* skeletal mass index, *CRP* C-reactive protein, *nPCR* normalized protein catabolic rate

### Clinical factors associated with sarcopenia

To examine the association between various clinical factors, including DM, and sarcopenia, multivariate regression analyses were performed (Table 2). In addition to age, duration of HD, BMI, and serum albumin levels, the presence of DM was determined to be an independent contributor to sarcopenia in patients undergoing HD (OR 3.11; 95% CI 1.63–5.93; *p* <  0.001).

**Table 2:** Odds ratios for clinical factors associated with the presence of sarcopenia

	OR	95% CI	p
Age (y)	1.08	1.05–1.11	< 0.001
Duration of HD (y)	1.10	1.04–1.16	< 0.001
Gender (male)	1.19	0.66–2.15	0.561
BMI (kg/m2)	0.73	0.65–0.83	< 0.001
Diabetes (yes)	3.11	1.63–5.93	< 0.001
Hemoglobin (g/dL)	1.11	0.86–1.42	0.421
Serum albumin (g/dL)	0.17	0.06–0.49	0.001
Log CRP	1.40	0.03–2.38	0.211

Results are from multivariate logistic regression analyses

Data are expressed as odds ratios: OR (95% confidential intervals: CI)

Abbreviations: *HD* hemodialysis, *BMI* body mass index, *CRP* C-reactive protein

### Subject outcomes

**Fig. 1:**
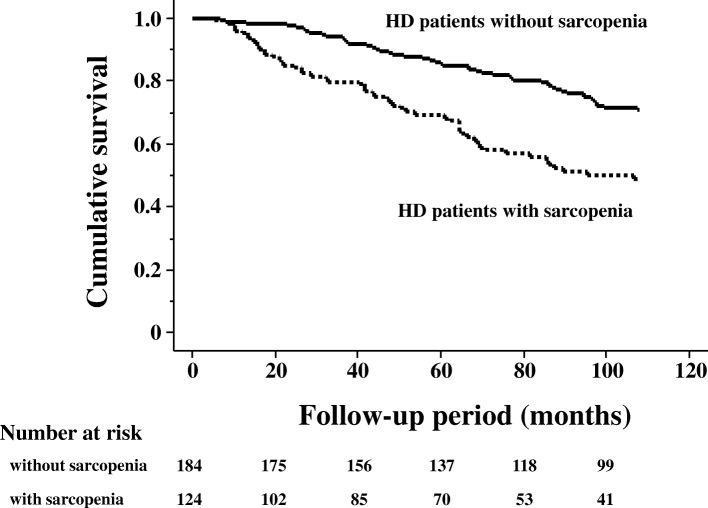
Mortality in patients undergoing hemodialysis with or without sarcopenia. Patients with sarcopenia showed significantly higher rates of all-cause mortality than those without sarcopenia (*p* <  0.001 using log-rank test)

The causes of the 100 deaths in the 308 patients included 35 fatal cardiovascular events: ischemic heart disease (IHD) (*n* = 10), congestive heart failure (*n* = 16), and stroke (*n* = 9). The 65 non-cardiovascular causes were infection (*n* = 22), malignancy (n = 10), uremia/cachexia (*n* = 7), sudden death (*n* = 5), liver cirrhosis (*n* = 4), others (*n* = 15) and unknown (n = 2). The Kaplan–Meier test was used to compare mortality between those with and without sarcopenia (Fig. 1). Patients with sarcopenia showed significantly higher all-cause mortality rates than those without sarcopenia (*p* <  0.001 using the log-rank test). Subsequently, multivariate Cox proportional hazards analyses were performed to confirm whether age, duration of HD, gender, BMI, presence of DM, serum albumin levels, presence of sarcopenia, Kt/V, and nPCR were independent predictors of mortality in patients with HD. Table 3 shows that the presence of DM was significantly associated with higher all-cause mortality (hazard ratio 2.39; 95% CI 1.51–3.81; *p* <  0.001), whereas the presence of sarcopenia was not.

**Table 3:** Hazard ratios for clinical factors associated with all-cause mortality

	HR	95% CI	p
Age (y)	1.09	1.06–1.11	< 0.001
Duration of HD (y)	1.03	0.99–1.07	0.214
Gender (male)	1.94	1.24–3.06	0.004
BMI (kg/m2)	1.00	0.92–1.08	0.910
Diabetes (yes)	2.39	1.51–3.81	< 0.001
Serum albumin (g/dL)	0.80	0.39–1.64	0.545
Sarcopenia (yes)	1.31	0.81–2.10	0.268
Kt/V	0.52	0.21–1.31	0.165
nPCR	0.59	0.20–1.75	0.339

Results are from multivariate Cox analyses

Data are expressed as hazard ratios: HR (95% confidential intervals: CI)

Abbreviations: *HD* hemodialysis, *BMI* body mass index, *nPCR* normalized protein catabolic rate

### Subgroup analyses according to age

Finally, the participants were divided into two groups: younger than 60 years (Age < 60) or 60 years and older (Age ≥ 60) (Additional file 1 Table S1). Similar statistical analyses were performed. The presence of DM independently contributed to sarcopenia in both groups (Additional file 2: Table S2). In younger group (Age < 60), there was no difference in mortality between patients with and without sarcopenia (Additional file 3: Figure S1 A). On the other hand, patients with sarcopenia showed significantly higher mortality rates than those without sarcopenia in older group (Age ≥ 60) (Additional file 3: Figure S1 B). In multivariate Cox proportional hazards analyses, the presence of DM was significantly associated with mortality in both group (Additional file 4: Table S3). Interestingly, the presence of sarcopenia was significantly associated with mortality only in older group (Age ≥ 60) (Additional file 4: Table S3).

## Discussion

We investigated the prevalence of sarcopenia in Japanese patients undergoing HD according to the AWGS criteria. The prevalence of sarcopenia was 40% in this study. In addition, we found that the presence of DM was an independent contributor to sarcopenia and an independent predictor of all-cause mortality in this population.

Compared with HS (muscle strength), the evaluation of muscle mass, which directly influences the prevalence of sarcopenia, is more problematic. Currently, DXA and bioimpedance analysis (BIA) have been the most commonly used methods for measuring muscle quantity. Unlike DXA, which has been used to evaluate body composition [14], the precision of BIA has been controversial, although it is inexpensive and non-invasive [2]. Therefore, DXA is still considered to be the more appropriate approach for the diagnosis of sarcopenia than BIA [2]. Another issue could be methods for normalizing muscle mass wherein different indexing methods, such as height squared, percentage of body weight, body surface area, and BMI have been used. In relation to this, Kittiskulnam et al. compared four different normalization methods and found considerable variations therein [15]. In this study, the presence of low muscle mass was defined as two standard deviations below normal mean of young adults. Even if the muscle mass was the same, the prevalence of low muscle mass indexed to height squared, percentage of body weight, body surface area, and BMI was 8.1, 25.3, 32.4, and 25.0%, respectively. Thus, a standard definition for sarcopenia is necessary for its accurate evaluation.

Major elements of sarcopenia include loss of muscle mass and reduced muscle strength. Although these elements are mutually related, recent findings suggest that both do not always diminish in parallel. Longitudinal observational studies have indicated that a decline in muscle strength seemed to precede a loss of muscle mass with age in healthy subjects [16, 17]. We have previously shown that poor muscle quality, defined as HS divided by arm muscle mass, was significantly associated with mortality in patients with HD [13]. Similarly, reports have shown that not only HS [18] but also lower extremity muscle strength [19] were significant predictors of all-cause mortality in patients undergoing dialysis, although muscle mass had not been evaluated in these studies. More importantly, a direct comparison between muscle mass or muscle strength and prognosis revealed that low muscle strength, rather than low muscle mass, was associated with mortality [5, 15]. Further studies will be necessary to determine which component of sarcopenia contributes more toward extending healthy life expectancy rather than mere survival.

In the current study, patients with DM had a significantly higher risk for sarcopenia as reported previously [8–10]. In addition to age, the presence of DM was a determining factor for all-cause mortality, which has a profound impact on the onset and progression of sarcopenia. Although the mechanism by which DM affects sarcopenia remains obscure, one possible explanation might be through the insufficient action of insulin. Insulin is known to be an anabolic hormone that can accelerate human skeletal muscle protein synthesis [20]. Aging is one of the strongest factors that reduce insulin-stimulated anabolic action, i.e., insulin resistance in human skeletal muscle [21], which is closely linked to DM. A recent report examined the association between various parameters of endogenous insulin secretion and sarcopenia evaluated through DXA in patients with type 2 DM [22]. In the aforementioned study, endogenous insulin secretion was significantly lower in patients with sarcopenia than in those without it, suggesting that insulin deficiency is an independent risk factor for sarcopenia. Bouchi et al. investigated the impact of insulin treatment on sarcopenia in patients with type 2 DM and found that the insulin-treated group had a significantly higher annual change in skeletal muscle mass than the non-insulin-treated group [23]. These findings suggest the critical role of insulin in DM-related sarcopenia.

Unexpectedly, no association of sarcopenia with mortality was found. However, stratified analyses according to age revealed that the presence of sarcopenia was an independent predictor of mortality in older group (Age ≥ 60). Although DM was involved in sarcopenia independent of age, the presence of DM might overwhelm the impact of sarcopenia on mortality in younger group (Age < 60). Indeed, DM was significantly associated with cardiovascular deaths in younger group, but not in older group (data not shown).

The strengths of the present study include the adaptation of the AWGS criteria and DXA for diagnosing sarcopenia, whereas limitations include its observational design and relatively small sample size. Moreover, we need to consider that DXA-measured lean mass is also influenced by the hydration status, which may lead to overestimation of muscle mass. Another serious limitation was the lack of precise data regarding DM, such as the type of DM, glycemic control status, and anti-diabetic treatment. In addition, we didn’t evaluate biological markers related to low-grade inflammation, oxidative stress, and advanced glycation end products. These factors may explain underlying mechanisms between sarcopenia and DM. Finally, we didn’t have complete data about nutritional status including diet intake, dialysis efficiency, dialysis adequacy, and history of comorbid diseases that could profoundly affect the prevalence of sarcopenia and mortality. Under our article submission, one interesting report has been published. Although the authors focused on the efficacy of L-carnitine supplementation for maintenance of both lean body mass and physical function, the prevalence of sarcopenia in Japanese patients undergoing HD was approximately 26–28% according to the AWGS criteria using DXA [24]. Further work is needed for definition and diagnosis of sarcopenia.

## Conclusions

The present study determined that the prevalence of sarcopenia among this cohort of patients undergoing HD was 40%. Moreover, the presence of DM was profoundly associated with sarcopenia morbidity and mortality in this population.

## Additional files

Additional file 1: **Table S1.** Characteristics of study participants according to age. (DOCX 18 kb)

Additional file 2: **Table S2.** Odds ratios for clinical factors associated with the presence of sarcopenia according to age. (DOCX 17 kb)

Additional file 3: **Figure S1.** Mortality in patients undergoing hemodialysis with or without sarcopenia according to age. In younger group (Age < 60), there was no difference in mortality between patients with and without sarcopenia (A). On the other hand, patients with sarcopenia showed significantly higher mortality rates than those without sarcopenia in older group (Age ≥ 60) (B). (PPT 612 kb)

Additional file 4: **Table S3.** Hazard ratios for clinical factors associated with all-cause mortality according to age. (DOCX 18 kb)

## Abbreviations

AWGS: Asian Working Group on Sarcopenia
BIA: bioimpedance analysis
CIs: confidence intervals
CKD: chronic kidney disease
CRP: C-reactive protein
DM: diabetes mellitus
DXA: dual-energy X-ray absorptiometry
HD: hemodialysis
HRs: hazard ratios
HS: handgrip strength
IQR: interquartile range
SD: standard deviation
SMI: skeletal mass index

## References

[1] Cruz-Jentoft AJ, Baeyens JP, Bauer JM, Boirie Y, Cederholm T, Landi F (2010). Sarcopenia: European consensus on definition and diagnosis: report of the European working group on sarcopenia in older people. Age Ageing.

[2] Chen LK, Liu LK, Woo J, Assantachai P, Auyeung TW, Bahyah KS (2014). Sarcopenia in Asia: consensus report of the Asian working group for sarcopenia. J Am Med Dir Assoc.

[3] Fahal IH (2014). Uraemic sarcopenia: aetiology and implications. Nephrol Dial Transplant.

[4] Kim JC, Kalantar-Zadeh K, Kopple JD (2013). Frailty and protein-energy wasting in elderly patients with end stage kidney disease. J Am Soc Nephrol.

[5] Isoyama N, Qureshi AR, Avesani CM, Lindholm B, Barany P, Heimburger O (2014). Comparative associations of muscle mass and muscle strength with mortality in dialysis patients. Clin J Am Soc Nephrol.

[6] Kim JK, Choi SR, Choi MJ, Kim SG, Lee YK, Noh JW (2014). Prevalence of and factors associated with sarcopenia in elderly patients with end-stage renal disease. Clin Nutr.

[7] Ren H, Gong D, Jia F, Xu B, Liu Z (2016). Sarcopenia in patients undergoing maintenance hemodialysis: incidence rate, risk factors and its effect on survival risk. Ren Fail.

[8] Park SW, Goodpaster BH, Strotmeyer ES, de Rekeneire N, Harris TB, Schwartz AV (2006). Decreased muscle strength and quality in older adults with type 2 diabetes: the health, aging, and body composition study. Diabetes..

[9] Kim TN, Park MS, Yang SJ, Yoo HJ, Kang HJ, Song W (2010). Prevalence and determinant factors of sarcopenia in patients with type 2 diabetes: the Korean Sarcopenic obesity study (KSOS). Diabetes Care.

[10] Wang T, Feng X, Zhou J, Gong H, Xia S, Wei Q (2016). Type 2 diabetes mellitus is associated with increased risks of sarcopenia and pre-sarcopenia in Chinese elderly. Sci Rep.

[11] American Diabetes A (2004). Diagnosis and classification of diabetes mellitus. Diabetes Care.

[12] Inaba M, Kurajoh M, Okuno S, Imanishi Y, Yamada S, Mori K (2010). Poor muscle quality rather than reduced lean body mass is responsible for the lower serum creatinine level in hemodialysis patients with diabetes mellitus. Clin Nephrol.

[13] Yoda M, Inaba M, Okuno S, Yoda K, Yamada S, Imanishi Y (2012). Poor muscle quality as a predictor of high mortality independent of diabetes in hemodialysis patients. Biomed Pharmacother.

[14] Shepherd JA, Ng BK, Sommer MJ (2017). Heymsfield SB.

[15] Kittiskulnam P, Chertow GM, Carrero JJ, Delgado C, Kaysen GA, Johansen KL (2017). Sarcopenia and its individual criteria are associated, in part, with mortality among patients on hemodialysis. Kidney Int.

[16] Hughes VA, Frontera WR, Wood M, Evans WJ, Dallal GE, Roubenoff R (2001). Longitudinal muscle strength changes in older adults: influence of muscle mass, physical activity, and health. J Gerontol A Biol Sci Med Sci.

[17] Goodpaster BH, Park SW, Harris TB, Kritchevsky SB, Nevitt M, Schwartz AV (2006). The loss of skeletal muscle strength, mass, and quality in older adults: the health, aging and body composition study. J Gerontol a-Biol.

[18] Vogt BP, Borges MC, Goes CR, Caramori JC (2016). Handgrip strength is an independent predictor of all-cause mortality in maintenance dialysis patients. Clin Nutr.

[19] Matsuzawa R, Matsunaga A, Wang G, Yamamoto S, Kutsuna T, Ishii A (2014). Relationship between lower extremity muscle strength and all-cause mortality in Japanese patients undergoing dialysis. Phys Ther.

[20] Fujita S, Rasmussen BB, Cadenas JG, Grady JJ, Volpi E (2006). Effect of insulin on human skeletal muscle protein synthesis is modulated by insulin-induced changes in muscle blood flow and amino acid availability. Am J Physiol Endocrinol Metab.

[21] Rasmussen BB, Fujita S, Wolfe RR, Mittendorfer B, Roy M, Rowe VL (2006). Insulin resistance of muscle protein metabolism in aging. FASEB J.

[22] Tanaka K, Kanazawa I, Sugimoto T (2015). Reduction in endogenous insulin secretion is a risk factor of sarcopenia in men with type 2 diabetes mellitus. Calcif Tissue Int.

[23] Bouchi R, Fukuda T, Takeuchi T, Nakano Y, Murakami M, Minami I (2017). Insulin treatment attenuates decline of muscle mass in Japanese patients with type 2 diabetes. Calcif Tissue Int.

[24] Maruyama T, Maruyama N, Higuchi T, Nagura C, Takashima H, Kitai M, et al. Efficacy of L-carnitine supplementation for improving lean body mass and physical function in patients on hemodialysis: a randomized controlled trial. Eur J Clin Nutr. (Epub ahead of print). 10.1038/s41430-018-0348-y 30353121

