# Supplementary material for: Impact of diabetes on sarcopenia and mortality in patients undergoing hemodialysis
Source: BMC Nephrol. 2019 Mar 28;20:105. doi: 10.1186/s12882-019-1271-8 (PMC6437886; doi:10.1186/s12882-019-1271-8)
Supplement: Supplementary file 1 — Table S1. Characteristics of study participants according to age. (DOCX 18 kb) [file 12882_2019_1271_MOESM1_ESM.docx]

Table S1. Characteristics of study participants according to age

|  | Age < 60 | Age ≥ 60 | p |
| --- | --- | --- | --- |
| Number of participants (%) | 171 (56%) | 137 (44%) |  |
| Gender (male/female) | 109 (35%)/62 (20%) | 76 (25%)/61 (20%) | 0.175 |
| Age (y) | 49.6 ± 7.5 | 68.6 ± 6.7 | < 0.001 |
| Duration of HD (y) | 7.7 ± 6.4 | 4.9 ± 5.1 | < 0.001 |
| BMI (kg/m2) | 20.7 ± 3.0 | 20.1 ± 2.5 | 0.052 |
| Diabetes (%) | 49 (16%) | 52 (17%) | 0.015 |
| Serum Albumin (g/dL) | 4.1 ± 0.3 | 3.9 ± 0.3 | < 0.001 |
| Serum Creatinine (mg/dL) | 12.2 ± 2.5 | 9.7 ± 2.2 | < 0.001 |
| Hemoglobin (g/dL) | 9.0 ± 1.3 | 8.8 ± 1.1 | 0.196 |
| HS (kg) | 26.6 ± 10.8 | 18.9 ± 8.2 | < 0.001 |
| SMI (kg/m2) | 6.04 ± 1.08 | 5.39 ± 0.88 | < 0.001 |
| CRP (mg/dL) | 0.15 (0.10-0.37) | 0.23 (0.10-0.70) | 0.021 |
| Kt/V | 1.16 ± 0.24 | 1.14 ± 0.28 | 0.656 |
| nPCR (g/kg per day) | 1.10 ± 0.22 | 0.97 ± 0.19 | < 0.001 |

Data are expressed as numbers, percentages, mean ± standard deviation, or median (interquartile range).

Abbreviations: HD, hemodialysis; BMI, body mass index; HS, handgrip strength; SMI, skeletal mass index; CRP, C-reactive protein; nPCR, normalized protein catabolic rate.
