# Supplementary material for: Impact of diabetes on sarcopenia and mortality in patients undergoing hemodialysis
Source: BMC Nephrol. 2019 Mar 28;20:105. doi: 10.1186/s12882-019-1271-8 (PMC6437886; doi:10.1186/s12882-019-1271-8)
Supplement: Supplementary file 2 — Table S2. Odds ratios for clinical factors associated with the presence of sarcopenia according to age. (DOCX 17 kb) [file 12882_2019_1271_MOESM2_ESM.docx]

Table S2. Odds ratios for clinical factors associated with the presence of sarcopenia according to age

**Age < 60**

|  | OR | 95% CI | p |
| --- | --- | --- | --- |
| Age (y) | 1.03 | 0.96-1.10 | 0.441 |
| Duration of HD (y) | 1.08 | 1.01-1.15 | 0.022 |
| Gender (male) | 1.09 | 0.46-2.57 | 0.845 |
| BMI (kg/m2) | 0.76 | 0.64-0.90 | 0.001 |
| Diabetes (yes) | 2.83 | 1.09-7.38 | 0.033 |
| Hemoglobin (g/dL) | 1.06 | 0.96-1.17 | 0.260 |
| Serum albumin (g/dL) | 0.11 | 0.02-0.47 | 0.003 |
| Log CRP | 1.63 | 0.76-3.52 | 0.210 |

**Age ≥ 60**

|  | OR | 95% CI | p |
| --- | --- | --- | --- |
| Age (y) | 1.09 | 1.01-1.16 | 0.020 |
| Duration of HD (y) | 1.18 | 1.07-1.31 | 0.001 |
| Gender (male) | 1.31 | 0.56-3.08 | 0.533 |
| BMI (kg/m2) | 0.67 | 0.57-0.83 | < 0.001 |
| Diabetes (yes) | 4.24 | 1.64-10.96 | 0.003 |
| Hemoglobin (g/dL) | 0.91 | 0.59-1.38 | 0.648 |
| Serum albumin (g/dL) | 0.24 | 0.05-1.10 | 0.067 |
| Log CRP | 1.15 | 0.54-2.47 | 0.721 |

Results are from multivariate logistic regression analyses.

Data are expressed as odds ratios: OR (95% confidential intervals: CI).

Abbreviations: HD, hemodialysis; BMI, body mass index; CRP, C-reactive protein.
