# Supplementary material for: Impact of diabetes on sarcopenia and mortality in patients undergoing hemodialysis
Source: BMC Nephrol. 2019 Mar 28;20:105. doi: 10.1186/s12882-019-1271-8 (PMC6437886; doi:10.1186/s12882-019-1271-8)
Supplement: Supplementary file 3 — Figure S1. Mortality in patients undergoing hemodialysis with or without sarcopenia according to age. In younger group (Age < 60), there was no difference in mortality between patients with and without sarcopenia (A). On the other hand, patients with sarcopenia showed significantly higher mortality rates than those without sarcopenia in older group (Age ≥ 60) (B). (PPT 612 kb) [file 12882_2019_1271_MOESM3_ESM.ppt]

## Slide 1
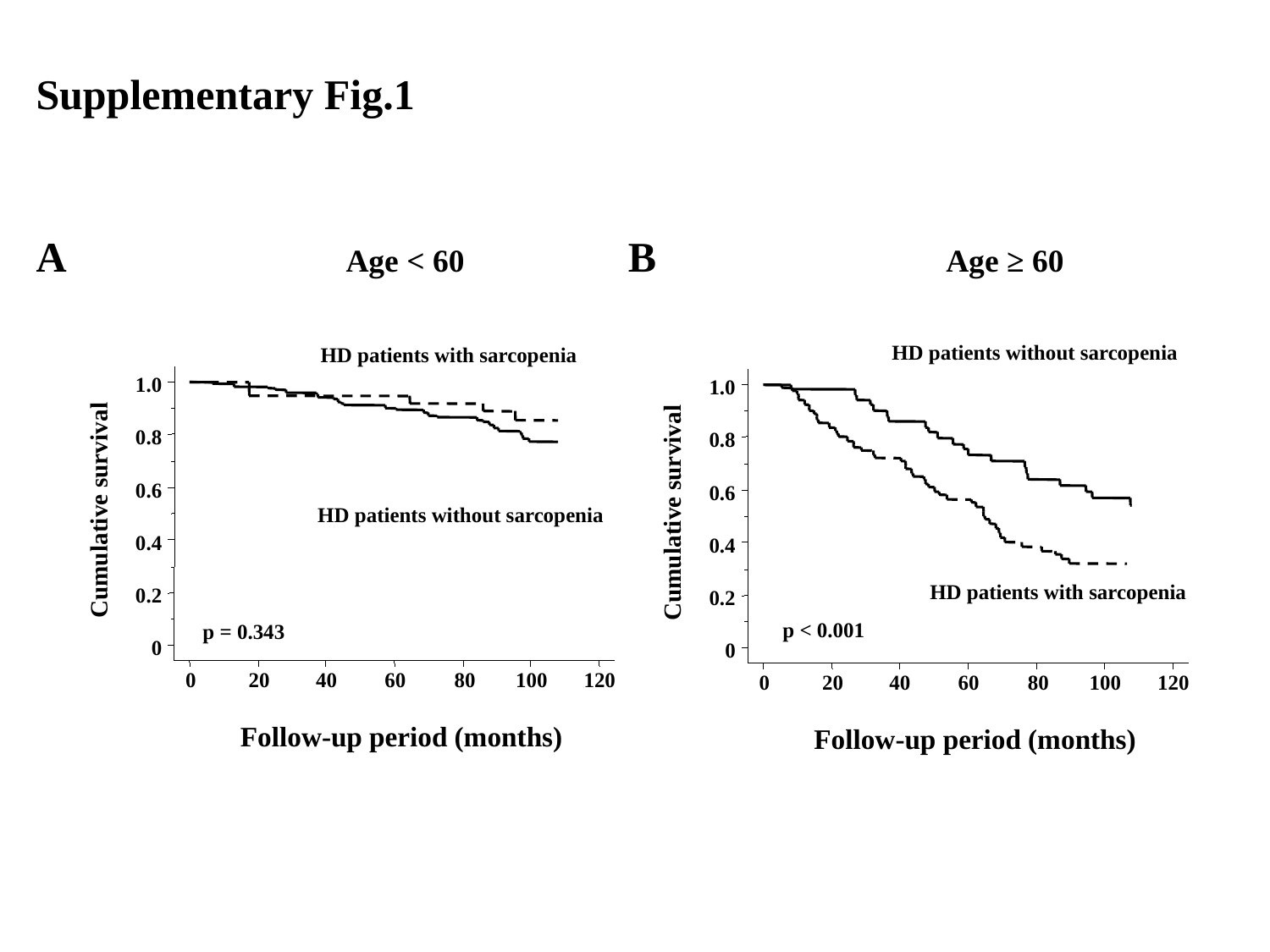

Supplementary Fig.1
A 	 　　　Age < 60
B	 　　　 Age ≥ 60
HD patients without sarcopenia
HD patients with sarcopenia
1.0
0.8
0.6
Cumulative survival
0.4
0.2
0
0
20
40
60
80
100
120
Follow-up period (months)
1.0
0.8
0.6
Cumulative survival
0.4
0.2
0
0
20
40
60
80
100
120
Follow-up period (months)
HD patients without sarcopenia
HD patients with sarcopenia
p < 0.001
p = 0.343
