# Supplementary material for: Impact of diabetes on sarcopenia and mortality in patients undergoing hemodialysis
Source: BMC Nephrol. 2019 Mar 28;20:105. doi: 10.1186/s12882-019-1271-8 (PMC6437886; doi:10.1186/s12882-019-1271-8)
Supplement: Supplementary file 4 — Table S3. Hazard ratios for clinical factors associated with all-cause mortality according to age. (DOCX 18 kb) [file 12882_2019_1271_MOESM4_ESM.docx]

Table S3. Hazard ratios for clinical factors associated with all-cause mortality according to age

**Age < 60**

|  | HR | 95% CI | p |
| --- | --- | --- | --- |
| Age (y) | 1.04 | 0.98-1.10 | 0.233 |
| Duration HD (y) | 1.10 | 1.03-1.17 | 0.006 |
| Gender (M) | 1.18 | 0.42-3.31 | 0.747 |
| BMI (kg/m^2^) | 0.95 | 0.82-1.11 | 0.534 |
| DM (yes) | 4.42 | 1.79-10.94 | 0.001 |
| Serum albumin (g/dL) | 1.21 | 0.33-4.45 | 0.779 |
| Sarcopenia (yes) | 0.42 | 0.15-1.19 | 0.103 |
| Kt/V | 0.54 | 0.05-6.39 | 0.626 |
| nPCR (g/kg/day) | 0.16 | 0.02-1.32 | 0.089 |

**Age ≥ 60**

|  | HR | 95% CI | p |
| --- | --- | --- | --- |
| Age (y) | 1.08 | 1.04-1.12 | < 0.001 |
| Duration HD (y) | 0.98 | 0.92-1.05 | 0.591 |
| Gender (M) | 2.07 | 1.24-3.47 | 0.006 |
| BMI (kg/m^2^) | 1.07 | 0.96-1.19 | 0.223 |
| DM (yes) | 1.95 | 1.10-3.46 | 0.023 |
| Serum albumin (g/dL) | 0.68 | 0.28-1.63 | 0.383 |
| Sarcopenia (yes) | 2.22 | 1.17-4.18 | 0.014 |
| Kt/V | 0.45 | 0.16-1.30 | 0.142 |
| nPCR (g/kg/day) | 0.85 | 0.21-3.42 | 0.820 |

Results are from multivariate Cox analyses.

Data are expressed as hazard ratios: HR (95% confidential intervals: CI).

Abbreviations: HD, hemodialysis; BMI, body mass index; nPCR, normalized protein catabolic rate.
